# Genotype^®^MTBDR*plus* and Xpert^®^MTB/RIF in the diagnosis of tuberculosis and resistant tuberculosis: cost analysis in a tertiary referral hospital

**DOI:** 10.1590/0037-8682-0175-2019

**Published:** 2020-02-07

**Authors:** Valéria Martins Soares, Isabela Neves de Almeida, Maria Cláudia Vater, Suely Alves, Lida Jouca de Assis Figueredo, Luciene Scherer, Afrânio Lineu Kritski, Wânia da Silva Carvalho, Silvana Spindola de Miranda

**Affiliations:** 1 Federação Hospitalar do Estado de Minas Gerais, Hospital Júlia Kubistchek, Laboratório de Microbiologia, Belo Horizonte, MG, Brasil.; 2 Universidade Federal de Minas Gerais, Laboratório de Pesquisa em Micobactérias, Belo Horizonte, MG, Brasil.; 3 Universidade Federal do Rio de Janeiro, Programa Acadêmico de Tuberculose, Rio de Janeiro, RJ, Brasil.; 4 Universidade Federal do Rio Grande do Sul, Porto Alegre, RS, Brasil.; 5 Universidade Federal de Minas Gerais, Faculdade de Farmácia, Belo Horizonte, MG, Brasil.; 6 Universidade Federal de Minas Gerais, Faculdade de Medicina, Grupo de Pesquisa em Micobactérias, Belo Horizonte, MG, Brasil.

**Keywords:** Tuberculosis, Mycobacteria, Diagnosis, Mean Cost, Activity Based Cost

## Abstract

**INTRODUCTION:**

The present study sought to assess the mean and activity based cost (ABC) of the laboratory diagnosis for tuberculosis through the application of conventional and molecular techniques-Xpert^®^MTB/RIF and Genotype^®^MTBDR*plus*-in a tertiary referral hospital in Brazil.

**METHODS:**

The mean cost and ABC formed the basis for the cost analysis of the TB laboratory diagnosis.

**RESULTS:**

The mean cost and ABC were US$ 4.00 and US$ 3.24, respectively, for a bacilloscopy; US$ 6.73 and US$ 5.27 for a Lowenstein-Jensen (LJ) culture; US$ 105.42 and US$ 76.56 for a drug sensitivity test (DST)-proportions method (PM) in LJ; US$ 148.45 and US$ 136.80 for a DST-BACTEC^TM^ MGIT^TM^ 960 system; US$ 11.53 and US$ 9.89 for an Xpert^®^MTB/RIF; and US$ 84.21 and US$ 48.38 for a Genotype^®^MTBDR*plus*.

**CONCLUSIONS:**

The mean cost and ABC proved to be good decision-making parameters in the diagnosis of TB and MDR-TB. The effective implementation of algorithms will depend on the conditions at each location.

## INTRODUCTION

Tuberculosis (TB) continues to be one of the primary public health issues in the world. Globally, 160,684 cases of multidrug-resistant (MDR) TB and rifampicin-resistant (RR) TB were detected and reported in 2017. Brazil is one of a group of 22 countries that account for 87% of all estimated TB cases around the world, and which the World Health Organization (WHO) lists as having high-priority health concerns^1^.

In 2017, 1,044 drug-resistant TB cases were diagnosed using Xpert^®^MTB/RIF (Cepheid, Sunnyvale, USA), by the proportions method in the Lowenstein Jensen (PM-LJ) or BACTEC^TM^ MGIT^TM^ 960 system (BD, Sparks, MD, USA)^2^. Following the implementation of Xpert^®^MTB/RIF, resistance diagnosis in Brazil improved, and in 2015, 63% of estimated MDR-TB cases were diagnosed, higher than the previous year’s rate of 40%^2^.

Since 2008, the WHO has endorsed the use of alternative molecular methods to detect TB and MDR-TB, which are designed for swifter TB diagnoses^3^. The costs of conventional and molecular methods have been studied in countries with both high and low prevalence of TB^4^ ^,^ ^5^. Local laboratories in Brazil have used molecular methods, such as Xpert^®^MTB/RIF, in their daily routines, while reference laboratories have used the Genotype^®^MTBDR*plus* test (Hain Lifescience, Nehren, Germany). These techniques have led to important findings concerning diagnostic accuracy. 

The Xpert^®^MTB/RIF test is able to detect both TB and RR through a polymerase chain reaction (PCR)^6^ ^,^ ^7^. The Genotype^®^MTBDR*plus* test detects the product enlarged by PCR through reverse hybridization. This technique presented satisfactory accuracy for the detection of *Mycobacterium tuberculosis*, as well as the detection of resistance to rifampicin and isoniazid, in many validation studies^8^ ^,^ ^9^, both in Brazil and worldwide^10^ ^,^ ^11^.

Although there are large numbers of studies on the accuracy of TB diagnostic tests, few studies have focused on cost, so developing cost-efficient methods is an important methodological field of enquiry^12^ - such as, for instance, activity based cost (ABC). 

ABC is appropriate for complex organizations such as hospitals because it improves managerial decisions, facilitates the determination of relevant costs, allows for the identification of actions geared towards reducing overhead costs, and provides greater precision in product costs. It also determines the costs of services and products, offers support during contract negotiations, helps customers understand cost reductions as a consequence of the use of their products and services, gives support for benchmarking, and determines the remainder of shared services^13^. 

In Brazil, a recent study attempted to provide subsidies for managers in order to identify their primary cost guidelines, as well as possible gains in efficiency and effectiveness, when adopting Xpert^®^MTB/RIF after implementing ABC methodology^14^. Another study assessed the mean cost and ABC of TB laboratory diagnoses by means of conventional techniques and the DetectTB^®^LabTest molecular kit in a high-complexity general hospital in a public health system^15^.

In this light, the present study assesses the mean cost and ABC of the laboratory diagnosis of TB and resistant TB in a tertiary referral hospital in the Brazilian Health System using Xpert^®^MTB/RIF, Genotype^®^MTBDR*plus*, as well as conventional techniques.

## METHODS

### Design and Study Site

This study collected primary data from the Microbiology Laboratory at Júlia Kubitschek Hospital (JKH) from January to December 2013. The JKH TB laboratory, which is a public and tertiary referral hospital for the treatment of TB and MDR-TB, conducts TB tests on patients who receive medical care at the hospital and provides educational and medical care activities in the state of Minas Gerais, Brazil.

### Laboratory Routine for TB Diagnosis and MDR-TB

JKH performed bacilloscopy without centrifugation by applying the Ziehl-Neelsen technique, the culture in the Lowenstein-Jensen (LJ) medium after decontamination by the Sodium Lauryl Sulfate method^16^, and the Xpert^®^MTB/RIF technique according to manufacturer instructions (Cepheid, Sunnyvale, USA). The State Reference Laboratory Ezequiel Dias Foundation (SRL/EDF) performed Drug Sensitivity Tests (DST) on antituberculosis drugs in a solid medium-LJ applying the proportions method (PM-LJ)^17^, or in a liquid medium using the MGIT (MGIT-DST) system. 

The Research Laboratory in Mycobacteria of the School of Medicine of the Federal University of Minas Gerais (RLM/SM/UFMG) performed the Genotype^®^MTBDR*plus* test, along with the Molecular Biology and Public Health Laboratory of the School of Pharmacy of Federal University of Minas Gerais (MBPH/SP/UFMG), according to manufacturer instructions (Hain Lifescience, Nehren, Germany).

Monthly, the JKH conducted an average of 300 bacilloscopies, 150 cultures, 15 Genotype^®^MTBDR*plus* tests, 15 DST (MGIT or PM) tests, and 150 Xpert^®^MTB/RIF tests on samples such as sputum, bronchoalveolar lavage, and traqueal aspirates, as well as extra-pulmonary samples such as cerebrospinal fluid, urine, and biopsies, among others.

This study was approved by the Research Ethics Committee of the Minas Gerais Hospital Foundation, logged under technical report number 018B/20, UFMG Ethics Committee protocol numbers CAAE-11821913.6.000.5257 and CAAE 0223.2412.7.1001.5149, and DEPE/HC protocol number 139/12. 

### Cost Analysis

The cost analysis of the TB laboratory diagnoses was based on two methods: mean cost and ABC. To calculate these costs, this study measured all direct and indirect costs involved in the process, including infrastructure, equipment, inputs, individual protection equipment (IPE), and human resources. It also included the maintenance of biosafety laboratory-2 (BSL-2) and two BSL-3 laboratories, including the SRL/EDF laboratory, by including the cost of the laboratories’ daily routines such as collection, transport, receipt, registration, processing, and release of results. The cost of each component was based on standard operating procedures for the specific performance of each technique. The mean cost and ABC were also calculated for the MGIT-DST and PM-LJ when considering only the JKH samples. These data were collected by consulting the purchasing, human resources, and maintenance sectors after receiving prior institutional authorization.

Mean cost was calculated by dividing the total cost by the quantity produced over a given period of time^13^, which in this study was one month. 

ABC was calculated using the activity, rather than the real consumed quantity, as the denominator for the calculation of unit cost per activity. This procedure aimed to avoid fluctuations in the calculation of an activity’s unit cost based on variations in the real processed quantities. The basic principle of this system was to quantify every item used in the process and considering the time necessary to complete the process^18^.

To calculate both costs (mean and ABC), this study measured all direct and indirect costs involved in the processes, including infrastructure, equipment, inputs, IPE, human resources, and the maintenance for BSL-2, the two BSL-3, and the SRL/EDF laboratory according to their daily routines during this study. The calculation of mean cost and ABC was also conducted for the MGIT-DST and PM-LJ, which considered only the JKH samples. These data were collected by consulting the purchasing, human resources, and maintenance sectors after receiving institutional authorization; the single health system table was not used.

All costs were expressed in U.S. dollars, using the conversion rate of US$ 1.00 = R$ 3.20 established by the Central Bank of Brazil in 2017^19^.

## RESULTS

Table 1 lists the mean cost and ABC of the tests assessed. For all of the diagnostic procedures analyzed, the mean costs were higher than the ABC. Among the phenotypic methods used for the DST, the ABC of the PM-LJ was US$ 76.56, compared to the cost of the MGIT-DST of US$ 136.80 (Table 1). 

**TABLE 1:** Mean and activity based cost of conventional and molecular tests.

Method	Samples/Month	Mean Cost	ABC
Bacilloscopy	300	US$ 4.00	US$ 3.24
LJ Culture	150	US$ 6.73	US$ 5.29
Xpert^®^MTB/RIF	150	US$ 11.53	US$ 9.89
Genotype^®^MTBDR*plus*	15	US$ 84.21	US$ 48.38
PM-LJ	84	US$ 105.42	US$ 76.56
MGIT-DST	40	US$ 148.45	US$ 136.80

Exchange rate of US$ 1.00 = R$ 3.20 in 2017 according to the Brazilian Central Bank.

The SRL/EDF performs 84 MGIT-DST and 40 PM-LJ per month. When calculating the mean cost of these methods considering only samples from JKH, the cost of the MGIT-DST increased from US$ 148.45 to US$ 244.32, while the PM-LJ cost increased from US$ 105.42 to US$ 169.36. The ABC did not change in this scenario. 

Table 2 lists the components of the ABC for each test performed to diagnose TB and MDR-TB. Upon performing the bacilloscopy, the human resources costs were what most increased the final cost. By contrast, for the phenotypic DST and molecular tests, the inputs affected the cost the most. 

**TABLE 2:** Cost components of ABC for each diagnostic test.

	Inputs	Human Resources	Equipment and Infrastructure	Total
Bacilloscopy	US$ 1.41 (43.5%)	US$ 1.68 (51.8%)	US$ 0.12 (3.7%)	US$ 3.24
LJ culture	US$ 2.52 (47.8%)	US$ 2.51 (47.6%)	US$ 0.26 (4.5%)	US$ 5.29
Xpert^®^MTB/RIF	US$ 8.62 (87.2%)	US$ 1.13 (11.4%)	US$ 0.13 (1.3%)	US$ 9.89
Genotype^®^MTBDR*plus*	US$ 29.36 (61%)	US$ 12.95 (27%)	US$ 6.07 (12%)	US$ 48.38
PM-LJ	US$ 63.04 (82%)	US$ 12.25 (16%)	US$ 1.27 (2%)	US$ 76.56
MGIT-DST	US$ 123.03 (90%)	US$ 11.04 (8%)	US$ 2.73 (2%)	US$ 136.80

Exchange rate of US$ 1.00 = R$ 3.20 in 2017 according to the Brazilian Central Bank

### Cost analysis of the diagnostic algorithms

Table 3 describes the diagnostic algorithms of the JKH laboratory diagnosis for negative and positive samples. The costs were higher for positive samples identified through culture than for negative samples, and a substantial increase in the cost was due to the introduction of phenotypic and molecular DST methods. It is noteworthy that in the hospital’s routine, Genotype^®^MTBDR*plus* was performed only when the cultures were positive, and the result was released, on average, 45 days before the phenotypic tests.

**TABLE 3:** Diagnostic algorithms for negative and positive samples in JKH’s routine.

Implementation of molecular tests with conventional tests-negative samples		
Algorithms	Mean cost	ABC
Xpert^®^MTB/RIF + LJ Culture	US$ 17.57	US$ 14.96
Bacilloscopy + Xpert^®^MTB/RIF + LJ culture	US$ 20.87	US$ 17.98
**Implementation of molecular tests with conventional tests-positive samples**		
**Algorithms**	Mean cost	ABC
Xpert^®^MTB/RIF + LJ culture + Genotype^®^MTBDR*plus* + PM-LJ	US$ 193.74	US$ 129.33
Xpert^®^MTB/RIF + LJ culture + Genotype^®^MTBDR*plus* + MGIT-DST	US$ 236.77	US$ 189.56
Bacilloscopy + Xpert^®^MTB/RIF + LJ culture + Genotype^®^MTBDR*plus* + PM-LJ	US$ 197.05	US$ 132.35
Bacilloscopy + Xpert^®^MTB/RIF + LJ culture + Genotype^®^MTBDR*plus* + MGIT-DST	US$ 240.07	US$ 192.59

Exchange rate of US$ 1.00 = R$ 3.20 in 2017 according to the Brazilian Central Bank.

The algorithms were performed in the order described in this table.

## DISCUSSION

The results of the mean cost and ABC proved to be of utmost importance in guiding policy-makers and laboratory managers in the structuring of the laboratory that performed both TB and MDR-TB diagnoses. This is especially important in relation to the Genotype^®^MTBDR*plus* test, which has a lower cost than the phenotypic tests. It is important to emphasize the incorporation of this fast, sensitive, and well-accepted method by the hospital’s clinical staff.

It should also be noted that ABC better reflects the reality of the cost analysis, as it provides the real value of the tests, as well as the importance of conducting cost studies based on data that has been duly computed, and not merely estimated based on other studies or the prices set by the Brazilian Health System^15^ ^,^ ^20^.

In the present study, the ABC of the bacilloscopy (US$ 3.24) and the mean cost (US$ 4.00) differed from those described in South Africa^4^, England^5^, and India^21^.

In South Africa, the ABC of the bacilloscopy was US$ 2.25, compared to US$ 2.38 in England^4^ ^,^ ^5^ and US$ 0.83 in India^21^. Among the series described in Brazil, another study found the ABC to be US$ 4.72 (Centrifuged Bacilloscopy) and US$ 4.15 (Direct Bacilloscopy)^15^. These differences could be due to the different methods used to calculate the cost, the characteristics of the organization, or the operation of the laboratories where the study was carried out.

The present study’s results demonstrate that the main component of the cost of a bacilloscopy was human resources, which is similar to that reported by the Brazilian Health Ministry^22^, given that both studies used methods that did not apply automated technologies.

The ABC of the LJ culture (US$ 5.27) was less than that reported by the Brazilian Health Ministry (US$ 9.59)^15^, in India (US$ 9.83)^21^, the cost reported in studies conducted by Almeida et al. 2017 (US$ 16.50), or in Africa, where there was a variation from US$ 12.16 to US$ 28.00^4^ ^,^ ^23^ ^,^ ^24^. These differences may be due to the use of different inputs, such as decontaminating agents and procedures, or the number of tubes used. In addition, the surveys were performed in different health care structures, which thwart comparisons.

Assessing the cost of the molecular tests, the ABC of the Xpert^®^MTB/RIF was US$ 9.89, while the mean cost was US$ 11.53. These results were similar to those of another Brazilian study where the ABC cost was US$ 11.11^14^, as well as those reported by Rupert et al. in India (US$ 12.29)^21^.

The high mean cost of the Genotype^®^MTBDR*plus* test can be explained by the relatively low number of tests performed (15 monthly tests). As the number of tests executed increases, this cost tends to diminish, which can justify concentration in reference laboratories that analyze larger quantities of samples. This flow has not yet been implemented in the daily routines of reference laboratories in Brazil, although the WHO has recommended it for the rapid detection of TB and MDR-TB^1^.

International studies have not evaluated the ABC of the Genotype^®^MTBDR*plus* test from a laboratory perspective; however, a study conducted in India that applied the bottom-up method, which is similar to the method used in this study, found a cost of US$ 18.18^21^, while the cost in the present study was US$ 48.38. This can be explained by the fact that this is a technology only commercialized by Biomerieux^®^, and by Biometrix^®^ in Brazil, and that different prices are commonly negotiated. It should be emphasized, as demonstrated by our study, that the inputs were the largest component of the total cost.

As regards the DST, the MGIT presented both a high mean cost (US$ 148.45) and a high ABC (US$ 136.80), given that the inputs proved to be the largest components of the cost (90%). The mean cost and the ABC for the PM were lower, but still higher than the molecular methods. Thus, in countries with few subsidies, one must assess the costs compared to other technologies endorsed by the WHO, such as the colorimetric redox indicator method, microscopically observed drug susceptibility, or a nitrate reductase assay^25^.

Upon comparing the identified costs with those covered by the Brazilian Unified Health System, the value paid for bacilloscopy (US$ 1.31)^22^ was 2.5 times less, while the value paid for the culture (US$ 1.76) was three times less. This difference shows the relevance of calculating ABC, as the horizontal view of this parameter allows for an analysis that is not restricted to profits, but rather to the real value of the cost chain. This view is based on planning and execution, and it aids in strategic decision-making, as well as in changes to the processes, waste elimination, and estimates drafting, based on the fact that the executed activities increase the efficiency of public services^26^.

Since the Brazilian Ministry of Health made the Xpert^®^MTB/RIF test available in the daily routines of some laboratories in Brazil without incorporating it into the Brazilian Unified Health System, what became relevant was the cost analysis of its use in distinct healthcare scenarios (primary, secondary, and tertiary) in different regions of the country. Regarding the ABC components of the Xpert^®^MTB/RIF, the inputs presented a greater share of the value (87.2%). Thus, the managers of the Brazilian Healthcare System must assess the economic impact of this new technology if this subsidy is eliminated^27^, considering that the test can be maintained in a laboratory’s daily routine. In this scenario, the costs of Xpert^®^MTB/RIF can increase to US$ 64.15 per sample, which is substantially more expensive than conventional methods. 

Since the Genotype^®^MTBDR*plus* and Xpert^®^MTB/RIF tests have not been made available or incorporated into the Brazilian Unified Health System, it is important to calculate the cost chain based on national data. Knowing the real costs involved in their execution can aid managers in their studies on economic impacts.

Given this context in the Brazilian Unified Health System, when deciding which algorithm to adopt, the managers of the healthcare system must bear in mind the specificities of the local healthcare system’s administration, as well as the economic situation of each region in the country. 

The present study does have some limitations. Salary costs and the average time of one’s work shift dedicated to TB diagnoses can vary geographically, as can the prices of inputs. Therefore, further studies are necessary in other regions of Brazil. Another limitation was in not evaluating cost-effectiveness, but this was not an aim of this study. Although the cost analysis did not directly include the effectiveness of the techniques conducted, its greatest advantage consisted of the facility of understanding its results, which were expressed in mean cost and ABC.

In conclusion, mean cost and ABC are good parameters for decision-making with regards to the diagnosis of TB and MDR-TB. When the mean cost is higher than the ABC, the method must be implemented in places with high demand. The effective implementation of these algorithms will depend on the conditions in each location.
